# Microbiome—pancreatic cancer crosstalk: From tumor biology to therapeutic intervention

**DOI:** 10.1002/imo2.70126

**Published:** 2026-07-29

**Authors:** Yuqin Xie, Jingyuan Ye, Sheng Wang, Maoyun Liu, Fengsheng Dai, Mingzhen Bai, Yi Wang, Athena Savoji, Yu Wang, Phillip B. Hylemon, Huiping Zhou, Hui Li, Dewei Li

**Affiliations:** ^1^ School of Medicine, Chongqing University Chongqing University Cancer Hospital Chongqing China; ^2^ Department of Hepatobiliary Pancreatic Tumor Center, Chongqing Key Laboratory for the Mechanism and Intervention of Cancer Metastasis Chongqing University Cancer Hospital Chongqing China; ^3^ Department of Chemical and Physical Sciences University of Toronto Mississauga Toronto Ontario Canada; ^4^ Department of Microbiology and Immunology Virginia Commonwealth University Richmond Virginia USA; ^5^ Stravitz‐Sanyal Institute for Liver Disease & Metabolic Health Virginia Commonwealth University Richmond VA USA; ^6^ Richmond Veteran Affairs Medical Center Richmond Virginia USA

**Keywords:** pancreatic cancer, intratumoral microbiome, tumor microenvironment, immune suppression, metabolic reprogramming, biomarkers, microbiota‐targeted therapy

## Abstract

Pancreatic cancer (PC) remains one of the most lethal malignancies worldwide, characterized by dense desmoplastic stroma, profound immunosuppression, and poor responsiveness to conventional therapies. Accumulating evidence positions the intratumoral microbiome, encompassing bacteria as well as understudied fungal and viral components, as a critical regulator of PC pathogenesis, tumor microenvironment (TME) remodeling, and therapeutic efficacy. In this review, we synthesize the current understanding of the pancreatic intratumoral microbiome, including its distinct composition relative to normal pancreatic tissue, gut microbiota, and oral microbiota. The PC intratumoral microbiome is characterized by enrichment of validated pro‐tumorigenic drivers (e.g., *Pseudomonas*, *Bacteroides* species) alongside protective taxa (e.g., *Lactobacillus* species). These taxonomic profiles vary significantly based on disease stage, patient demographics, and treatment history. We dissected the mechanisms by which microbes and their derivatives drive PC initiation and progression, including immune suppression through modulation of myeloid‐derived suppressor cells and regulatory T cells, metabolic reprogramming involving short‐chain fatty acids and the tryptophan metabolite 3‐indoleacetic acid (3‐IAA), and stromal remodeling via activation of cancer‐associated fibroblasts. The influence of the microbiome on therapeutic response is examined, with particular emphasis on microbial‐mediated mechanisms that undermine treatment efficacy. We further summarize diagnostic and prognostic biomarkers, including salivary microbial signatures with moderate sensitivity and specificity, circulating microbial DNA, and composite microbial risk scores that integrate multiple bacterial species to predict survival. Therapeutic strategies targeting the microbiome are discussed, distinguishing clinically translatable approaches from experimental concepts requiring further validation. Finally, we outline challenges in standardizing profiling methods, validating microbial signatures across diverse populations, and translating preclinical insights to clinical practice, emphasizing the need for multi‐omics integration and interdisciplinary collaboration to advance microbiome‐driven PC management.

## Highlights

- The pancreatic intratumoral microbiome originates primarily via ductal, hematogenous, and biliary translocation routes.
- Intratumoral microbes drive pancreatic oncogenesis through interconnected mechanisms of immune suppression, metabolic reprogramming, and desmoplastic stroma remodeling.
- Microbial species and their metabolites significantly compromise the efficacy of standard chemotherapy and immunotherapy.
- Microbiota‐targeted strategies, including precision antibiotics, fecal microbiota transplantation, and engineered microbes, represent emerging avenues to overcome therapeutic resistance, though most remain at preclinical stages.

## Graphical abstract

The intratumoral microbiome acts as a critical regulator in pancreatic cancer progression and therapeutic resistance. This comprehensive review delineates the origins of pancreatic microbes, elucidates their multifaceted mechanisms in driving immune suppression, metabolic reprogramming, and stromal desmoplasia, and highlights the clinical potential of microbiota‐targeted diagnostic and therapeutic strategies.

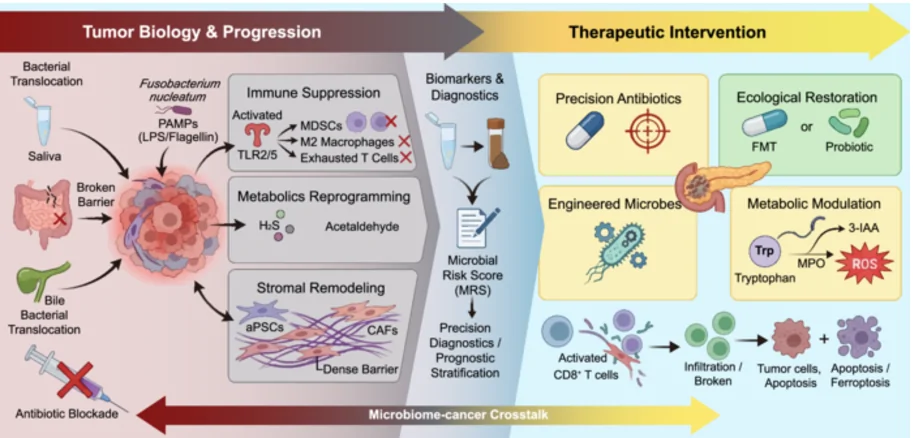

## 1 INTRODUCTION

Pancreatic cancer (PC) remains one of the most lethal malignancies and ranks among the leading causes of cancer‐related mortality worldwide [1, 2]. Because symptoms are often insidious, the majority of patients present at advanced stages when curative resection is no longer feasible, underscoring the dire need for diagnostic strategies to detect PC at an early, resectable stage [3, 4]. Recent studies highlight the promise of integrating molecular biomarkers and imaging for early detection [5, 6]. Its dismal prognosis stems from late diagnosis, inherent therapeutic resistance, and a highly immunosuppressive tumor microenvironment, with a 5‐year survival rate below 10% even in developed countries [7, 8, 9]. The TME of PC is uniquely characterized by a dense, fibrotic stroma composed of cancer‐associated fibroblasts, extracellular matrix proteins, and immune cells that collectively create a physical barrier to drug delivery and suppress antitumor immune responses [10, 11]. Tumor‐associated macrophages, myeloid‐derived suppressor cells, and regulatory T cells dominate the immune landscape, while effector T cells and natural killer cells are scarce and functionally impaired [12].

Over the past decade, the human microbiome has emerged as a critical environmental factor shaping cancer biology across multiple organ systems [13, 14]. The gut microbiome has been linked to colorectal, liver, and breast cancers, but the intratumoral microbiome has gained increasing attention for its direct interactions with cancer cells and the TME [15, 16]. This microbial community resides within the tumor parenchyma and stroma [17, 18]. It is not isolated to the pancreas but exhibits complex connections with microbial communities at other body sites, highlighting the existence of interconnected microbial axes that influence disease progression [19, 20].

Comparative analyses of oral, intestinal, and pancreatic samples from PC patients reveal 73 shared amplicon sequence variants, pointing to microbial translocation or shared ecological drivers [21]. Salivary profiles of PC patients further reflect this cross‐talk, exhibiting distinct taxonomic shifts relative to healthy controls [21, 22]. These shifted oral taxa show consistent associations with pancreatic tumor microbiomes, supporting the use of oral samples as non‐invasive surrogates for pancreatic microbial profiling, particularly given the limited accessibility of pancreatic tissue in early disease stages [22, 23].

Early studies using 16S rRNA gene sequencing and fluorescence in situ hybridization demonstrated that cancerous pancreatic tissue harbors a more abundant and distinct microbiome than normal pancreatic tissue in both mice and humans [24]. Subsequent work linked microbial dysbiosis to key PC hallmarks: immune suppression, stromal expansion, and therapeutic resistance [24]. Fungal taxa such as *Candida albicans* have been associated with carcinogenesis through acetaldehyde production and pro‐inflammatory signaling, though direct causal evidence in PC remains limited [25, 26, 27]. The roles of vitals and bacteriophages remain understudied, but emerging evidence suggests they may modulate bacterial communities or directly interact with cancer cells to promote oncogenesis [28, 29]. Large‐scale studies in early‐stage resected PC have identified tumor microbial signatures associated with patient survival, including a microbial risk score integrating 11 bacterial species that predicts overall survival across multiple cohorts [30].

Host and environmental factors—including sex, smoking, dietary patterns, and prior antibiotic exposure—further shape the pancreatic microbiome and its functional impacts, underscoring the need to account for these variables in microbiome research and clinical practice [24, 31, 32, 33, 34, 35, 36, 37, 38, 39, 40, 41].

This review integrates preclinical and clinical data to characterize the pancreatic intratumoral microbiome, define its mechanistic roles in PC initiation and progression, and highlight its potential for clinical translation. By synthesizing findings from metagenomic sequencing, animal models, and early‐phase clinical trials, we aim to provide a comprehensive framework for understanding the microbiome's role in PC and identify avenues for future research that may improve patient outcomes.

## 2 COMPOSITION AND ORIGINS OF THE PANCREATIC INTRATUMORAL MICROBIOME

### Taxonomic composition

PC tumors harbor a distinct microbial community with reduced α‐diversity compared to normal pancreatic tissue, reflecting a state of dysbiosis that accompanies malignant transformation [40, 42, 43]. At the phylum level, Proteobacteria, Bacteroidetes, and Firmicutes dominate human PC samples, collectively accounting for over 95% of the microbial community [42, 44]. Proteobacteria are the most abundant phylum, comprising approximately 45% of the intratumoral microbiome, followed by Bacteroidetes (31%) and Firmicutes (22%). Actinobacteria are also universally present, albeit at lower levels (1%), while other phyla, such as Synergistetes and Euryarchaeota, are detected at negligible abundances but show significant enrichment in PC patients compared to healthy controls [24].

This phylum‐level profile differs sharply from the gut microbiome, where Proteobacteria constitute only approximately 8% of the community, and *Prevotella* and *Bacteroides* species predominate. These taxonomic discrepancies underscore the specificity of the intratumoral microbiome and suggest that microbial translocation to the pancreas is a selective process rather than random dissemination. The gut microbiome is dominated by Firmicutes and Bacteroidetes, with Proteobacteria representing a minor component, further supporting the idea that the pancreatic intratumoral microbiome is not a passive reflection of gut microbial composition but a distinct community shaped by the unique microenvironmental conditions of the pancreas [24, 40, 42].

At the genus level, *Pseudomonas* and *Elizabethkingia* are highly enriched in PC tumors, distinguishing them from normal pancreatic tissue [24, 42, 45]. Among *Pseudomonas* species, *Pseudomonas aeruginosa* is a common pathogenic taxon, but it is also a soil and water bacterium that can easily contaminate clinical samples—highlighting the importance of precise species identification in microbiome profiling [43, 46, 47]. *Pseudomonas* species are particularly prevalent, with some studies reporting detection in over 70% of PC specimens, and their abundance correlates with advanced disease stage and poor prognosis [24, 40, 42]. *Fusobacterium nucleatum* is a cross‐site taxon linked to immunomodulation and tumor progression [48, 49, 50]. Recent work adds *Bacteroides fragilis* and *Bacteroides thetaiotaomicron* to this list of clinically relevant taxa, as producers of 3‐IAA—a metabolite associated with favorable chemotherapy responses [51].

Anaerobic taxa including *Lactobacillus* and *Peptoniphilus* correlate with poor outcomes, while a signature including *Pseudoxanthomonas*, *Streptomyces*, *Saccharopolyspora*, and *Bacillus clausii* predicts long‐term survival [42, 52]. Intraductal papillary mucinous neoplasms (IPMNs), precursor lesions to PC, exhibit a higher relative abundance of Bacteroidetes and Fusobacteria in invasive compared to noninvasive lesions, suggesting microbial involvement in malignant progression [53, 54]. The basal‐like subtype of PC, characterized by high invasiveness and poor prognosis, is associated with distinct microbial signatures that promote epithelial–mesenchymal transition, further highlighting subtype‐specific microbiome heterogeneity [24, 42, 55].

Pushalkar et al. [24] demonstrated that murine models of PC recapitulate key features of the human intratumoral microbiome. Specifically, KC mice (Ptf1aCre, *LSL‐KrasG12D*) show progressive enrichment of Actinobacteria (reaching approximately 60% abundance by Week 20) and Deferribacteres (abruptly increasing in Weeks 28–36) compared to wild‐type mice. They also identified *Bifidobacterium pseudolongum* as the most abundant *Bifidobacterium* species in late‐stage pancreatic dysplasia in KC mice, suggesting a role in disease progression. Additionally, KPC mice (*Pdx1Cre*, *LSL‐KrasG12D*, *Trp53R172H*) with aggressive tumors exhibit distinct microbial profiles with altered Bacteroidetes and Firmicutes abundances compared to those with slow progression. These data confirm dynamic taxonomic shifts during PC development, with stage‐specific changes that may reflect evolving microenvironmental conditions and host–microbe interactions.

Beyond bacteria, the pancreatic intratumoral microbiome includes fungal and viral components that remain understudied but functionally relevant [45, 56, 57]. The mycobiome, or fungal community, is dominated by *Candida* species in PC tumors, with *C. albicans* being the most frequently detected taxon [45, 57, 58]. Additionally, *Malassezia* species have been implicated in PC progression, with increased abundance observed in invasive compared with noninvasive IPMNs [59] and correlation with protumorigenic inflammation via activation of the mannose‐binding lectin (MBL) pathway and C3 complement cascade [45]. *C. albicans* has been associated with pancreatic carcinogenesis, potentially through production of acetaldehyde and nitrosamines, which may contribute to DNA damage and epithelial dysplasia [26, 60]. However, direct causal evidence in human PC remains to be established.

Fungal dysbiosis has been associated with malignant transformation of pancreatic precursor lesions, with increased abundance of *Candida* and *Malassezia* species observed in invasive compared with noninvasive IPMNs. The virome, encompassing viruses and bacteriophages, remains poorly characterized in PC [28, 61, 62]. Bacteriophages could potentially influence the TME through selective lysis of bacterial populations or promotion of horizontal gene transfer, though functional evidence in PC is currently lacking [28]. Emerging evidence also suggests that tumor‐associated eukaryotic viruses, such as certain herpesviruses, may directly interact with cancer cells to modulate immune evasion pathways and promote oncogenesis, though their specific roles in PC remain to be fully defined [56, 63, 64]. Technical limitations in sequencing low‐abundance viral and fungal taxa have hindered comprehensive profiling, but advances in metagenomic approaches are enabling deeper characterization of these non‐bacterial components [65]. Their inclusion in future studies is critical for understanding the full complexity of the intratumoral microbiome and its impact on PC biology [40, 42, 65].

### The bidirectional gut‐pancreas axis and translocation

The pancreatic intratumoral microbiome originates primarily from the gut and oral cavity, with microbial translocation occurring via multiple routes [66, 67]. As illustrated in Figure 1, the pancreatic intratumoral microbiome primarily originates from the gut and oral cavity through multiple translocation routes, including the pancreatic duct, hematogenous spread, and the biliary tract. Gut‐pancreatic translocation is facilitated by the pancreatic duct, which maintains anatomical continuity with the intestinal tract, thereby providing a direct pathway for microbial migration [68, 69]. Disruption of the intestinal epithelial barrier induced by inflammation, chemotherapy, or oncogenic *KRAS* signaling facilitates bacterial translocation into the systemic circulation and subsequent colonization of the pancreas [70, 71, 72]. Oncogenic *KRAS* signaling, a driver mutation in over 90% of PC cases, alters gut epithelial permeability by modulating tight junction proteins, promoting the translocation of pathobionts that contribute to pancreatic inflammation and tumorigenesis [40, 73].

**Figure 1:**
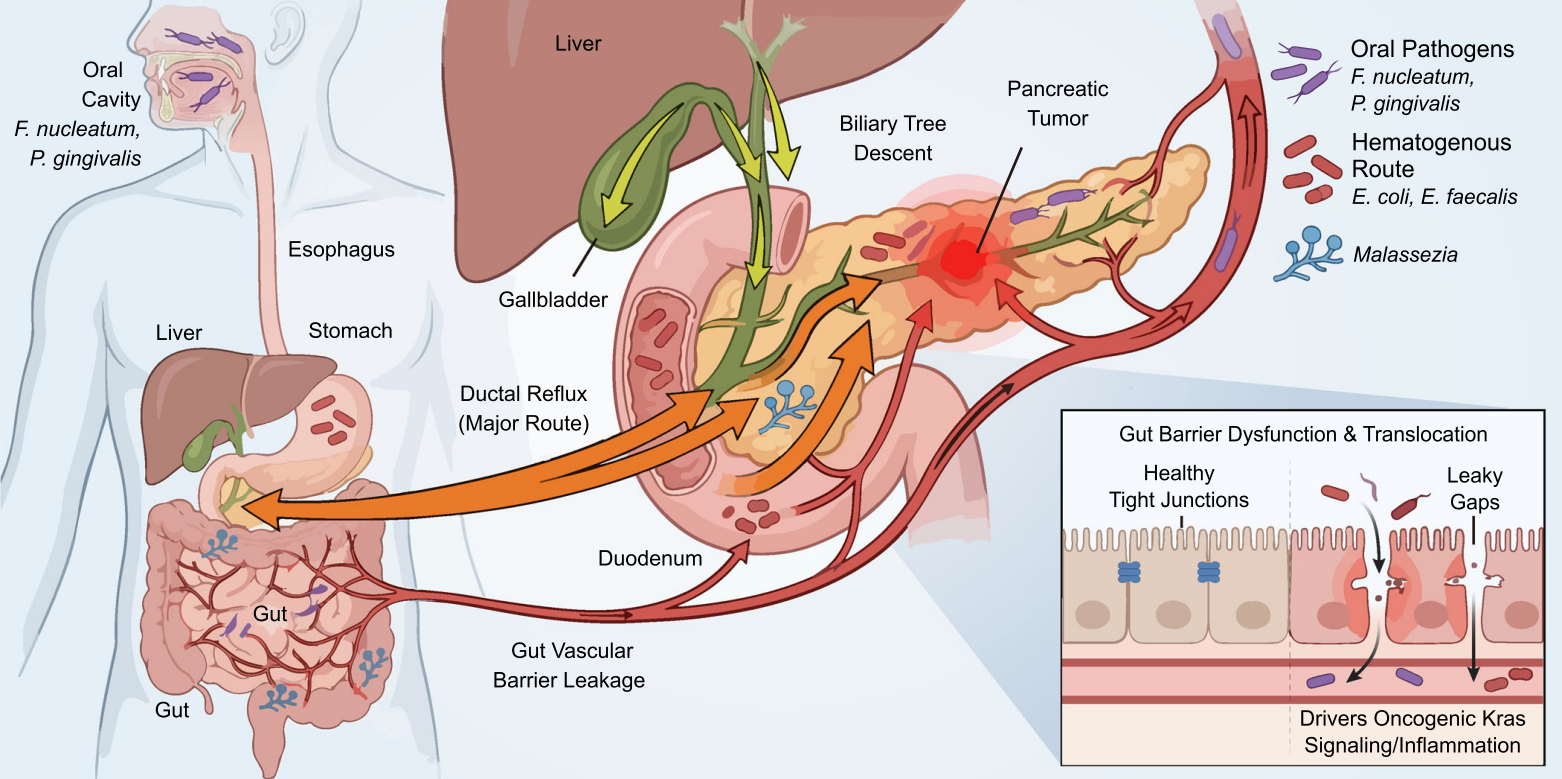
Origins and translocation routes of the intratumoral microbiome in pancreatic cancer. Intratumoral microbes mainly originate from the oral cavity and the intestinal tract and access the pancreatic tumor microenvironment through three major routes: (yellow arrows) the ductal route, involving retrograde migration of duodenal bacteria through the pancreatic duct; (red arrows) the hematogenous route, characterized by systemic dissemination facilitated by intestinal barrier disruption, often driven by oncogenic *KRAS* signaling, and increased tumor vascular permeability; and (green arrows) the biliary route, whereby microbes translocate from the biliary tree into pancreatic tissue. Notably, this process is bidirectional: pancreatic exocrine dysfunction promotes gut dysbiosis, which further compromises intestinal barrier integrity and facilitates additional microbial dissemination to the pancreas.

Studies in wild‐type mice have demonstrated that orally administered fluorescently labeled *Enterococcus faecalis* and GFP‐labeled *Escherichia coli* migrate to the pancreas, confirming direct gut‐pancreatic microbial translocation. Repopulation experiments in antibiotic‐treated wild‐type mice show that the gut microbiome from KPC mice has a higher capacity for translocation to the pancreas compared to the wild‐type gut microbiome, indicating that cancer‐associated gut dysbiosis enhances microbial dissemination [24, 74]. These findings suggest that the gut microbiome of PC patients may be inherently more prone to translocate to the pancreas, creating a feed‐forward loop that promotes tumor progression.

Oral microbes reach the pancreas via the gastrointestinal tract or systemic circulation [20, 67, 75]. Periodontal pathogens such as *Porphyromonas gingivalis* have been detected in pancreatic tumor tissues, where they drive tumorigenesis via secretion of neutrophil elastase—a protease that promotes inflammation and tissue remodeling [76]. Salivary microbiome studies have reinforced this oral‐pancreatic link, with oral taxa showing consistent associations with pancreatic tumor microbiomes and PC risk [23, 67, 77]. *F. nucleatum* and *Gemella morbillorum* are among the shared taxa identified between oral and pancreatic tissues, supporting the hypothesis of oral‐pancreatic microbial translocation [21].

Beyond unidirectional translocation, the gut‐pancreatic axis operates bidirectionally [78, 79]. Pancreatic exocrine dysfunction, common in PC and chronic pancreatitis, impairs nutrient digestion and absorption by reducing pancreatic enzyme secretion [80]. This creates a gut microenvironment enriched for proinflammatory taxa that further enhance intestinal barrier disruption. This resulting dysbiosis promotes additional microbial translocation to the pancreas, reinforcing the protumorigenic cycle [81]. Biliary tract dysfunction, which is frequently associated with PC, can modulate the gut microbiota by altering bile acid composition, as bile acids function as signaling molecules that regulate microbial growth and host immune responses [82, 83]. This bidirectional crosstalk highlights the interconnectedness of pancreatic health and gut microbial homeostasis, with disruptions in one compartment exacerbating dysregulation in the other.

Other potential routes of microbial entry into the pancreas include the biliary tract and hematogenous spread [84, 85]. Notably, PC often metastasizes to the liver, but whether specific microbes are associated with liver metastasis (and if their profiles differ from primary tumors) remains understudied. Future studies should compare microbial communities between primary PC tumors and liver metastases to identify metastasis‐specific microbial drivers, particularly given the role of conjugated bile acids in accelerating liver metastatic progression via S1PR2 signaling [86]. The bile microbiome of PC patients exhibits an altered composition compared to benign pancreaticobiliary diseases, with lower abundance of *Escherichia* and increased *Streptococcus*, suggesting that biliary microbes may contribute to the intratumoral community [87, 88]. Hematogenous spread is supported by the detection of oral and gut microbes in the systemic circulation of PC patients, with subsequent colonization of the pancreas facilitated by the leaky vasculature of the tumor microenvironment [89].

### Host and environmental correlates

Host demographic and lifestyle factors shape the composition and functional capacity of the pancreatic intratumoral microbiome, [90, 91] as illustrated in Figure 2. Sex is a key modifier, with male PC patients exhibiting a more protumorigenic microbial community [90, 92]. This sex‐specific difference may be mediated by hormonal factors, as androgens modulate gut barrier function and microbial composition [93, 94]. Male patients also experience a worse prognosis compared to females, highlighting the clinical relevance of sex‐specific microbiome‐host interactions [90, 95]. Age further shapes the pancreatic microbiome: older adults exhibit reduced microbial diversity and altered taxonomic composition, which may contribute to increased PC risk with age [78, 96]. Chronic pancreatitis, a major PC risk factor, is associated with pancreatic and gut microbial dysbiosis characterized by increased proinflammatory taxa [78, 97, 98]. Individuals with familial PC syndromes or genetic variants in immune signaling and gut barrier function genes may also exhibit inherent vulnerabilities to dysbiosis that accelerate malignant transformation [99, 100, 101].

**Figure 2:**
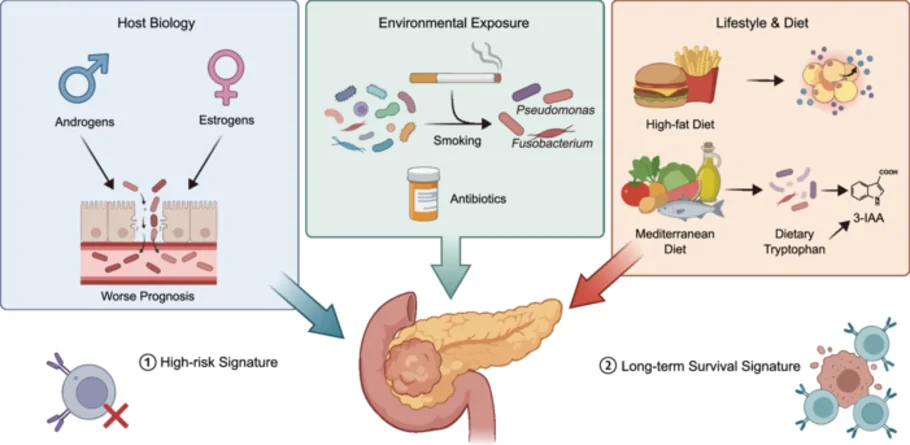
Host, lifestyle, and environmental determinants of the pancreatic cancer‐associated microbiome. The composition and functional capacity of the intratumoral microbiome are shaped by a range of systemic host‐related factors. Host biology influences microbial recruitment and tumor‐associated signatures, as sex‐specific hormonal profiles, such as androgens, modulate gut permeability and correlate with increased intratumoral *Fusobacterium* abundance, with male patients often exhibiting more pro‐tumorigenic microbial patterns. Environmental exposures further remodel the microbiome: cigarette smoking reduces microbial diversity and enriches tumor‐promoting taxa, while prior antibiotic use depletes beneficial taxa and compromises subsequent treatment responses. Lifestyle and dietary factors also play a critical role, with high‐fat diets promoting proinflammatory dysbiosis, whereas fiber‐rich Mediterranean diets support beneficial microbes that generate antitumor metabolites, including 3‐indoleacetic acid (3‐IAA). Specifically, dietary tryptophan metabolism enhances chemosensitivity. Furthermore, a high‐risk microbial signature is characterized by low diversity, high pathogen abundance, and an immune‐cold microenvironment leading to poor survival, contrasting with long‐term survival signatures driven by high diversity and beneficial species like *Lactobacillus*.

Smoking is another major environmental factor influencing the pancreatic microbiome [90, 102]. Smokers show altered microbial composition, with increased abundance of tumor‐promoting taxa and reduced diversity compared to nonsmokers [103]. Smoking cessation may partially reverse these microbial changes, but the long‐term impact on the pancreatic microbiome and PC risk remains unclear [102, 104]. Chronic alcohol consumption, a known risk factor for pancreatitis and PC, similarly disrupts intestinal barrier integrity and promotes translocation of gut microbes into the pancreas [78, 105].

Diet plays a critical role in shaping the gut‐pancreatic microbiome axis [106, 107]. High intake of oily or fatty foods is associated with microbial dysbiosis and increased proinflammatory taxa [108, 109], while the Mediterranean diet, rich in prebiotic fibers, may reduce tumor burden through beneficial microbiome modulation [110, 111, 112]. Dietary tryptophan serves as a precursor for microbial metabolites, including 3‐IAA, directly linking dietary intake to microbial metabolic activity relevant to PC [51, 113]. Prior antibiotic use disrupts the pancreatic microbiome and impairs responses to immunotherapy and chemotherapy [114, 115]. These findings underscore the need to account for antimicrobial exposure in microbiome research and to exercise caution when prescribing antibiotics to PC patients, particularly those undergoing immunotherapy or chemotherapy.

## 3 MECHANISMS LINKING INTRATUMORAL MICROBIOTA TO PANCREATIC ONCOGENESIS

The intratumoral microbiome is associated with PC development through diverse, interconnected mechanisms. Critically, most current evidence is correlative—distinguishing causal relationships from associative findings remains a fundamental challenge that must be acknowledged when interpreting the studies discussed below. The multifaceted mechanisms by which the microbiome shapes the PC tumor microenvironment, including immune evasion, metabolic reprogramming, and desmoplastic expansion, are illustrated in Figure 3. The microbiome promotes oncogenesis mainly via three interconnected mechanisms. Immune Suppression: Activation of *TLR*2/5 pathways recruits MDSCs and polarizes macrophages toward a tolerogenic M2 phenotype, leading to CD8^+^ T‐cell exhaustion. Metabolic Reprogramming: Microbes produce oncometabolites such as hydrogen sulfide (H_2_S) and acetaldehyde, which induce DNA damage and genomic instability. Conversely, microbe‐derived 3‐IAA can enhance therapeutic efficacy via MPO‐mediated ROS production. Stromal Remodeling: Microbial signals activate pancreatic stellate cells and CAFs, driving dense collagen deposition and desmoplasia. While many microbial taxa and metabolites are linked to PC hallmarks, only a subset have been validated as functional drivers through complementary experimental approaches: germ‐free models, microbial ablation/reconstitution, targeted metabolite manipulation, and single‐cell functional validation [24, 42, 116]. These approaches help distinguish causal drivers from opportunistic passengers that colonize the altered TME. In the following sections, we describe these mechanisms with particular emphasis on causal evidence where available. Table 1 provides a systematic summary of representative intratumoral microbes and their validated mechanistic roles in PC progression, including immune evasion, metabolic reprogramming, and stromal remodeling.

**Figure 3:**
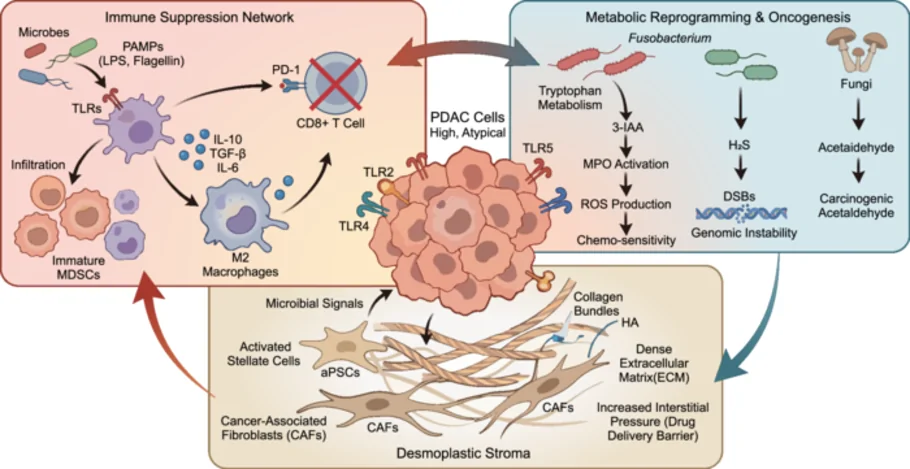
Integrated mechanisms of microbiome‐driven remodeling of the tumor microenvironment. Microbes shape the pancreatic cancer tumor microenvironment through three interconnected core processes: immune suppression, metabolic reprogramming, and stromal remodeling, with extensive crosstalk between modules to drive tumor progression and therapeutic resistance. Microbe‐derived pathogen‐associated molecular patterns (PAMPs) from *Fusobacterium nucleatum* and other pathobionts, such as lipopolysaccharide (LPS) and flagellin, activate Toll‐like receptors (*TLR*2/5), leading to the recruitment of myeloid‐derived suppressor cells (MDSCs) and polarization of macrophages toward a tolerogenic M2 phenotype, ultimately promoting CD8^+^ T‐cell exhaustion. Fungal taxa including *Malassezia* spp. and *Candida albicans* further drive immune tolerance via activation of the MBL‐C3 complement cascade, IDO‐mediated tryptophan metabolism, and regulatory T‐cell (Treg) recruitment. Metabolic reprogramming arises from microbe‐derived oncometabolites, including hydrogen sulfide (H_2_S) from *F. nucleatum* and acetaldehyde from *C. albicans*, which induce DNA damage and genomic instability. Notably, microbial‐derived 3‐indoleacetic acid (3‐IAA) from *Bacteroides* species enhances chemotherapy efficacy via myeloperoxidase (MPO)‐mediated reactive oxygen species (ROS) production in tumor cells, while the short‐chain fatty acid butyrate exerts context‐dependent dual effects, inducing tumor cell ferroptosis while also modulating regulatory T‐cell function. Stromal remodeling is driven by microbial signals including *TLR*9 ligation and bacterial extracellular vesicles, which activate pancreatic stellate cells and cancer‐associated fibroblasts (CAFs), resulting in dense collagen deposition and desmoplasia that forms a physical barrier to drug delivery and immune cell infiltration.

**Table 1:** Representative intratumoral microbes and their roles in pancreatic cancer progression.

Taxons	Metabolites	Molecular mechanisms	Impact on pancreatic cancer	Evidence level	Therapeutic implications
*Fusobacterium nucleatum*	H_2_S	Inhibiting DNA repair enzymes, inducing DNA damage	Promoting oncogenesis and genomic instability	Strong Correlative	Metronidazole‐mediated depletion; H_2_S production inhibitors
*F. nucleatum*	*TLR4*/*MYD88* signaling	Inducing autophagy, reducing tumor immunogenicity	Leading to immune ICI resistance	Strong Correlative	Metronidazole + anti‐PD‐1/PD‐L1 combination
*Pseudomonas*	CDA	Converting gemcitabine into inactive dFdU	Inducing chemoresistance and associated with poor prognosis	Causal	Ciprofloxacin + gemcitabine combination; CDA inhibitors
*Candida albicans*	Acetaldehyde	Inducing DNA mutations and activating NF‐κB pathway	Promoting carcinogenesis and epithelial dysplasia	Correlative	Antifungal therapy (requires PC‐specific validation)
*Bacteroides* spp.	3‐IAA	Activating neutrophil MPO, increasing intratumoral ROS	Enhancing chemotherapy efficacy	Causal	Time‐limited tryptophan supplementation to boost 3‐IAA/MPO axis
*Bifidobacterium pseudolongum*	Inosine	Activating T‐cell A2A receptors and promoting polarization of macrophages	Enhancing ICI therapeutic response	Strong Correlative	Probiotic supplementation combined with anti‐PD‐L1
*Porphyromonas gingivalis*	Neutrophil elastase	Driving chronic inflammation and tissue remodeling	Promoting tumor initiation and progression	Causal	Periodontal intervention; neutrophil elastase inhibitors

Abbreviations: 3‐IAA, indole‐3‐acetic acid; CDA, cytidine deaminase; ICI, checkpoint inhibitor; MPO, myeloperoxidase; *MYD88*, myeloid differentiation primary response 88; ROS, reactive oxygen species; *TLR* 4, toll‐like receptor 4.

### Immune suppression

The intratumoral microbiome is a central driver of PC's immunosuppressive TME, modulating both innate and adaptive immune responses to promote tumor progression [24, 42] (Figure 3). Causal evidence for this role comes from bacterial ablation studies in preclinical KC mouse models. Antibiotic treatment or germ‐free conditions lead to immunogenic reprogramming of the TME, including a reduction in myeloid‐derived suppressor cells and an increase in M1 macrophage differentiation [107]. This reprogramming promotes TH1 differentiation of CD4^+^ T cells and CD8^+^ T‐cell activation, directly demonstrating the microbiome's causal role in suppressing antitumor immunity [24].

Mechanistically, the intratumoral microbiome induces a tolerogenic immune program by differentially activating select toll‐like receptors on monocytic cells [24]. *TLR*2 and *TLR*5 ligation enhances PC progression by promoting innate and adaptive immune suppression [117]. *TLR*5 activation by bacterial flagellin increases myeloid‐derived suppressor cell infiltration and M2 macrophage polarization, while reducing CD8^+^ T‐cell cytotoxicity [118]. Inhibition of *TLR* signaling by blocking TRAF6 abrogates the tumor‐promoting effects of the PC microbiome, confirming that *TLR*‐dependent pathways are critical for microbial‐mediated immune suppression [24, 116].

Fungal taxa may also contribute to immune suppression. *C. albicans* has been shown, primarily in nonpancreatic models, to act through IDO‐mediated tryptophan degradation and *Candida* lysin‐induced proinflammatory signaling, potentially inducing tolerogenic dendritic cells and regulatory T cells [119]. The relevance of these mechanisms to human PC requires further investigation. Additionally, *Malassezia* spp. activates the MBL pathway and C3 complement cascade [45], promoting protumorigenic inflammation and type 2 immunity via IL‐33 secretion and ILC2 recruitment [58]. Dendritic cells exposed to fungal components show reduced capacity to present antigens and activate T cells, while regulatory T cells secrete IL‐10 and TGFβ to inhibit effector T‐cell function [120, 121].

Tumor‐associated macrophages are key mediators of microbial‐induced immune suppression in PC [122, 123]. Cell‐free extracts from gut bacteria of PC‐bearing hosts reduce MHC II expression and upregulate IL‐10 in splenic macrophages, impairing their antigen‐presenting capacity [24]. *B. pseudolongum*, a taxon enriched in the pancreatic microbiome of KC mice, polarizes macrophages toward a tolerogenic phenotype, mitigating M1 differentiation and T‐cell activation. These microbiome‐entrained macrophages suppress T‐cell immunogenicity by reducing CD44 and PD‐1 expression on CD4^+^ and CD8^+^ T cells, preventing TH1 differentiation. Conversely, bacterial ablation restores macrophage antigen‐presenting function, leading to enhanced intratumoral T‐cell activation and reduced tumor burden [24].

Bacterial extracellular vesicles (BEVs) from gut bacteria further modulate macrophage function [124, 125, 126]. *Helicobacter pylori*‐derived BEVs induce M2 macrophage polarization and secretion of proinflammatory cytokines such as TNF‐α and IL‐6, reinforcing immune suppression [127]. These vesicles transport virulence factors and microbial metabolites to macrophages, activating signaling pathways that promote tolerogenic polarization [125, 128].

Recent studies link the pancreatic tumor microbiome to specific immune cell subsets [24, 31, 42]. Fecal microbiota transplantation (FMT) from healthy donors to colorectal cancer mice reduces *Foxp3*+ regulatory T‐cell accumulation and increases infiltration of CD8^+^ T cells and CD49b^+^ natural killer cells, enhancing antitumor immune responses [129]. In PC, the microbial risk score derived from 11 survival‐associated bacteria correlates with reduced memory B‐cell infiltration and increased naive CD4^+^ T‐cell presence, potentially via modulation of B‐cell receptor and T‐cell receptor signaling pathways [30]. *P. gingivalis* in pancreatic tumors correlates with reduced chemical complementarity between bacterial antigens and tumor‐resident T‐cell receptors, impairing adaptive immune recognition [130].

### Metabolic reprogramming

The intratumoral microbiome modulates PC metabolism through production of oncometabolites, alteration of nutrient availability, and competition with cancer cells for essential nutrients [43, 131]. Microbial metabolites such as short‐chain fatty acids, secondary bile acids, hydrogen sulfide, and trimethylamine N‐oxide have been implicated in PC progression, with causal roles supported by metabolite supplementation and inhibition studies [82, 132].

*F. nucleatum*, enriched in the intratumoral microbiome of PC, employs dual pro‐tumorigenic mechanisms: it produces hydrogen sulfide in response to red meat intake [92, 133, 134], which inhibits DNA repair enzymes and induces genomic instability [135, 136, 137]. Additionally, its surface protein Fap2 binds to Gal‐β(1‐3)‐N‐acetyl‐d‐galactosamine overexpressed on PC cells, facilitating bacterial colonization and immune suppression. This binding mechanism, conserved from colorectal cancer, is critical for *F. nucleatum*‐mediated PC progression [138]. This causal relationship is supported by experiments showing that reducing *F. nucleatum* abundance or inhibiting hydrogen sulfide production reduces tumor burden in murine models [139, 140]. *C. albicans* metabolizes ethanol and glucose to produce acetaldehyde, a carcinogen that induces DNA mutations and epithelial dysplasia [141]. Acetaldehyde also enhances inflammation by activating NF‐κB signaling, further promoting tumor progression [142].

Short‐chain fatty acids, particularly butyrate, exhibit context‐dependent dual roles in PC that are dictated by concentration, spatial localization, and immune context [143, 144, 145]. This duality represents one of the major unresolved questions in the field. On one hand, intratumoral butyrate induces ferroptosis in PC cells via superoxidative stress and intracellular lipid accumulation, and inhibits histone deacetylases to promote apoptosis [142, 144, 145]. On the other hand, systemic butyrate can enhance regulatory T‐cell function and limit anti‐CTLA‐4 efficacy in the gut [146]. These opposing effects underscore a critical knowledge gap: the conditions under which butyrate shifts from tumor‐suppressive to tumor‐promoting remain poorly defined. Resolving this paradox—through spatially resolved metabolomics and cell‐type‐specific knockout models—is essential before butyrate‐based interventions can be rationally deployed in PC patients [146, 147, 148].

Conversely, *Clostridium butyricum* and its metabolite butyrate promote ferroptosis susceptibility in PC cells by inducing superoxidative stress and intracellular lipid accumulation [149]. These findings highlight the context‐dependent metabolic effects of microbial metabolites, which may be either pro‐tumorigenic or antitumorigenic depending on the cellular and molecular environment [150].

Trimethylamine N‐oxide, derived from dietary choline and l‐carnitine by gut bacteria, modulates pancreatic metabolism by inhibiting reverse cholesterol transport and promoting foam cell formation via *CD36*/MAPK signaling [151, 152, 153]. TMAO also activates the NLRP3 inflammasome and ROS‐TXNIP pathway, leading to the secretion of IL‐1β and IL‐18, which further enhances inflammation and immune suppression [154].

A key metabolic mechanism involves 3‐IAA, produced by *B. fragilis* and *B. thetaiotaomicron* [155, 156, 157]. This metabolite accumulates in chemotherapy‐responsive patients and enhances treatment efficacy by activating neutrophil‐derived myeloperoxidase (MPO). MPO oxidizes 3‐IAA to generate 3‐methylene‐2‐oxindole (MOI), [51] which downregulates glutathione peroxidases (GPX3, GPX7) to increase reactive oxygen species in cancer cells, impairing metabolic adaptation and proliferation [51]. Given that global neutrophil infiltration in PC typically drives immune evasion via MDSCs and TANs, broadly enhancing neutrophil recruitment is counterproductive [158, 159]. Instead, exploiting the 3‐IAA/MPO axis by reprogramming existing intra‐tumoral neutrophils to maximize local MPO activity offers a highly targeted, host‐directed therapeutic strategy [51, 160, 161].

### Stromal remodeling

The intratumoral microbiome plays a crucial role in shaping the dense desmoplastic stroma of PC, which is composed of cancer‐associated fibroblasts, extracellular matrix proteins, and immune cells [162]. This stroma acts as a physical barrier to therapeutic delivery and antitumor immunity, making stromal remodeling a key mechanism by which the microbiome influences PC progression [116, 163]. Causal evidence comes from studies utilizing germ‐free KC murine models, which show significantly reduced stromal expansion compared to conventionally raised mice; repopulation with PC‐derived microbiota restores stromal fibrosis, directly demonstrating the microbiome's role in stromal development [42].

Mechanistically, microbiome‐induced *TLR* signaling in cancer‐associated fibroblasts and pancreatic stellate cells promotes the secretion of pro‐fibrotic cytokines and extracellular matrix proteins. *TLR*9 ligation in pancreatic stellate cells enhances tumorigenesis by inducing pro‐inflammatory cytokines and stromal expansion [164]. These pro‐fibrotic cytokines, including TGFβ and IL‐1β, activate cancer‐associated fibroblasts to secrete collagen and fibronectin, increasing extracellular matrix deposition and stromal density [165, 166, 167, 168]. This stromal remodeling enhances tumor cell invasion and protects cancer cells from chemotherapy and immune‐cell attack [169, 170].

BEVs from gut bacteria further contribute to stromal remodeling [171, 172, 173]. *B. fragilis*‐derived BEVs induce cancer‐associated fibroblast activation via E‐cadherin cleavage and IL‐8 production, promoting extracellular matrix deposition [174, 175]. These vesicles transport virulence factors and microbial metabolites to cancer‐associated fibroblasts, activating signaling pathways that enhance their pro‐fibrotic phenotype [171, 176, 177].

Recent studies link microbial communities to stromal heterogeneity in PC [24, 30, 42, 45, 162]. Intratumoral bacteria such as *Alcaligenes faecalis* correlate with stromal gene expression patterns associated with poor prognosis [30, 52, 178, 179]. In contrast, FMT from long‐term PC survivors to mice reduces stromal density and enhances immune infiltration, highlighting the potential of microbial modulation to reverse stromal fibrosis [42]. Targeting the microbiome may therefore reduce stromal density and improve therapeutic accessibility, enhancing the efficacy of chemotherapy and immunotherapy [81, 180].

A critical consideration in interpreting microbiome‐PC interactions is distinguishing causal drivers—microbes that actively promote tumorigenesis—from passengers—microbes that colonize tumors opportunistically due to the altered TME [42, 181]. While many associations have been reported, causal evidence is strongest for taxa and mechanisms validated through complementary approaches. These include germ‐free animal models, microbial ablation or reconstitution, and targeted manipulation of microbial metabolites or signaling pathways [24, 42, 182, 183].

For example, *P. gingivalis* is an established causal driver: its depletion reduces tumor burden in murine models and reintroduction restores pro‐tumorigenic phenotypes [184]. Similarly, microbial metabolites such as hydrogen sulfide and 3‐IAA have direct effects on cancer cell biology, supporting their causal role. In contrast, some taxa may be passengers, benefiting from the nutrient‐rich, immunosuppressive TME without actively contributing to tumor progression [51, 157, 185, 186].

Disentangling these relationships is challenging due to the complexity of microbial communities and host‐microbe interactions [187, 188]. However, emerging technologies such as single‐cell sequencing, spatial transcriptomics, and engineered microbial consortia are enabling more precise validation of causal mechanisms [189, 190, 191]. Future studies must prioritize experimental approaches that establish causality to avoid overinterpreting correlative data, which is critical for translating microbial insights into effective therapies [192, 193].

## 4 MICROBIOTA‐MEDIATED IMPAIRMENT OF PC THERAPY

The intratumoral microbiome undermines chemotherapy, immunotherapy, and targeted therapy efficacy through diverse mechanisms [194, 195], as summarized in Figure 4A,B. This section focuses on microbial contributions to treatment failure, establishing the rationale for therapeutic strategies discussed in Section 6.

**Figure 4:**
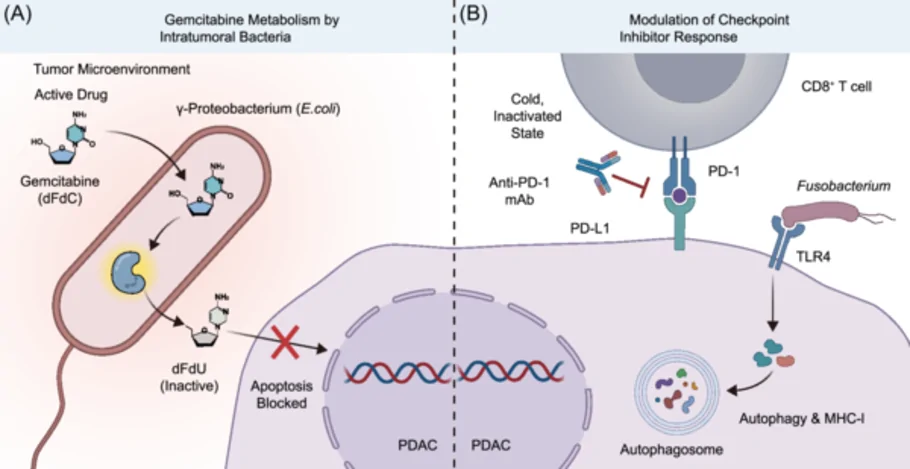
Microbial mechanisms underlying therapeutic resistance in pancreatic cancer. Intratumoral bacteria compromise standard‐of‐care therapies through multiple mechanisms. (A) γ‐proteobacteria expressing cytidine deaminase (CDA) enzymatically inactivate gemcitabine (dFdC) by converting it into the inactive metabolite dFdU, thereby attenuating DNA damage and apoptosis in tumor cells. (B) Pathobionts such as *Fusobacterium nucleatum* impair immunotherapy efficacy by inducing *TLR4*‐dependent autophagy and major histocompatibility complex class I (MHC‐I) degradation, resulting in CD8^+^ T‐cell dysfunction and resistance to immune checkpoint blockade. Together, these mechanisms highlight the multifaceted role of the microbiome in shaping therapeutic resistance and toxicity in pancreatic cancer.

### Chemotherapy resistance and toxicity

Conventional chemotherapeutic agents for PC are frequently compromised by microbial activity, leading to reduced efficacy and increased toxicity [162]. Gemcitabine, a first‐line nucleoside analog, is directly inactivated by γ‐proteobacteria‐expressing cytidine deaminase [75, 196, 197]. This enzyme converts gemcitabine to its inactive form, 2′,2′‐difluorodeoxyuridine, preventing the drug from inhibiting DNA synthesis in cancer cells [196, 198]. Clinical studies confirm that high intratumoral γ‐proteobacteria abundance correlates with poor response to gemcitabine‐based chemotherapy, establishing this microbial community as a key driver of treatment failure [41, 196]. Biliary stent placement and neoadjuvant chemotherapy further exacerbate this issue by increasing intratumoral colonization by Enterobacteriaceae, a family of γ‐proteobacteria that amplifies gemcitabine inactivation [198].

Oxaliplatin, a platinum‐based agent, relies on gut microbiota to exert its antitumor effects, creating a paradox: while microbial depletion reduces oxaliplatin‐induced DNA damage and tumor regression, certain microbial taxa may also promote resistance [199, 200]. The gut microbiome primes intratumoral myeloid cells for reactive oxygen species production via NOX2, which enhances oxaliplatin‐induced DNA damage [199]. However, microbial dysbiosis can disrupt this priming, reducing drug efficacy [200, 201]. Additionally, oxaliplatin‐induced immunogenic cell death, which is critical for durable antitumor immunity, depends on intact microbial communities. Bacterial ablation impairs the activation of antitumor T‐cell responses, thereby limiting the curative potential of the drug [202].

Microbiome‐mediated irinotecan toxicity limits therapeutic dosing [200, 203]. The detoxified hepatic metabolite SN‐38G is excreted into the gut, where bacterial β‐glucuronidases reconvert it to the active SN‐38 form, causing severe intestinal damage and diarrhea [204, 205, 206, 207, 208]. This microbial‐mediated toxicity affects up to 80% of patients treated with irinotecan, reducing treatment adherence and quality of life without improving antitumor efficacy [209].

Chemotherapy itself exacerbates microbial dysbiosis, creating a feed‐forward loop that further impairs treatment outcomes [208, 210, 211]. Chemotherapeutic agents disrupt the gut epithelial barrier, promoting microbial translocation and increasing the abundance of pro‐inflammatory taxa such as Enterobacteriaceae [72, 212]. This dysbiosis enhances systemic inflammation and contributes to gastrointestinal toxicity, including mucositis and diarrhea, which are major limiting factors of chemotherapy in PC [72, 211]. Broad‐spectrum antibiotic use during chemotherapy further disrupts microbial balance, depleting beneficial taxa that support gut barrier function and immune regulation, while failing to target the specific pathogens driving resistance [213].

### Immunotherapy resistance

Immune checkpoint inhibitors (ICIs) show limited efficacy in PC, with response rates below 10%, largely due to the profoundly immunosuppressive TME shaped by the intratumoral microbiome (detailed mechanisms of immune suppression are discussed in Section 3) [214, 215] (Figure 4A,B). Critically, bacterial ablation in PC models upregulates PD‐1 on intratumoral T cells, enabling synergistic anti‐PD‐1 efficacy [24]. This implies the microbiome actively suppresses PD‐1 expression to prevent ICI‐mediated T‐cell reinvigoration, establishing a direct mechanistic link between the intratumoral microbiome and ICI failure [196, 216].

Specific taxa actively drive immunoresistance; for example, *F. nucleatum* induces protective autophagy via TLR4/MYD88 signaling, which clinically correlates with reduced anti‐PD‐1/PD‐L1 response and shorter survival [33, 48, 217, 218, 219].

Depletion of beneficial taxa such as *Bifidobacterium* and *Akkermansia muciniphila* further impairs ICI efficacy by reducing dendritic cell maturation and CD8 + T‐cell activation [39, 216, 220, 221, 222]. FMT from ICI‐responsive donors restores anti‐PD‐1 response in preclinical models, confirming the microbiome's causal role in immunotherapy outcomes [221, 223, 224].

### Targeted therapy limitations

Targeted therapies for PC, including tyrosine kinase inhibitors (TKIs) and PARP inhibitors, are also influenced by the intratumoral microbiome, which can reduce drug activity or promote resistance [139, 196, 225, 226]. PARP inhibitors induce CD8^+^ T‐cell recruitment via the cGAS/STING pathway, but microbial dysbiosis impairs this immune activation by reducing innate immune signaling [227, 228, 229]. In *BRCA*‐deficient PC models, microbial depletion reduces the synergistic efficacy of PARP inhibitors and ICIs, highlighting the microbiome's role in enhancing targeted therapy responses [24, 221, 230].

TKIs targeting the VEGF/VEGFR pathway, such as sunitinib and cabozantinib, reduce immunosuppressive cell infiltration in the TME, but this effect is blunted by microbial dysbiosis [199, 231, 232, 233]. Beneficial microbes enhance TKI‐mediated immune cell redistribution, while protumorigenic taxa promote the persistence of myeloid‐derived suppressor cells and regulatory T cells, limiting drug efficacy [114, 191, 234, 235]. Whether microbiota‐modulating strategies can enhance TKI efficacy in PC remains an open question requiring dedicated preclinical investigation [236, 237, 238, 239].

Microbial metabolites may also directly interfere with targeted therapy mechanisms [240, 241]. Short‐chain fatty acids and trimethylamine N‐oxide modulate signaling pathways targeted by TKIs, including MAPK and PI3K/Akt, potentially reducing drug activity [150, 242, 243]. Bacterial dysbiosis alters the expression of genes involved in targeted therapy resistance, such as those encoding drug efflux pumps and DNA repair enzymes, further compromising treatment outcomes [244, 245, 246]. These microbiome‐mediated effects highlight the need to consider the microbiome when developing and administering targeted therapies for PC [24, 225, 247, 248, 249].

## 5 DIAGNOSTIC AND PROGNOSTIC POTENTIAL

### Diagnostic biomarkers

A multi‐modal landscape of microbiological biomarkers, spanning oral, fecal, biliary, and tissue sources, provides a non‐invasive framework for PC screening and survival prediction (Figure 5A–C). Early detection of PC remains a major clinical challenge, as symptoms are often absent or nonspecific in the early stages [250, 251, 252]. Microbial biomarkers offer a promising noninvasive approach to complement existing imaging and serological tests, which lack sufficient sensitivity and specificity for early diagnosis [37, 225].

**Figure 5:**
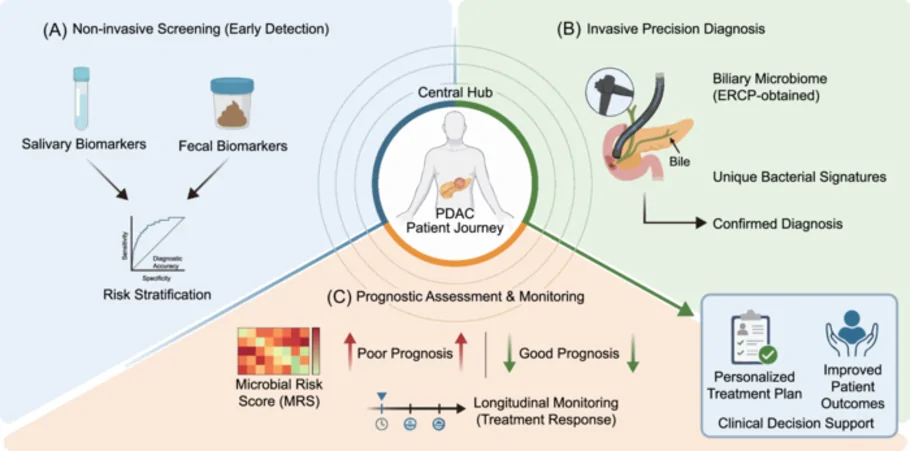
Clinical applications of microbiome‐based biomarkers in pancreatic cancer. Microbiome‐derived biomarkers span noninvasive and invasive sampling modalities, and can be integrated across the patient journey to support early detection, differential diagnosis, prognostic risk stratification, and personalized treatment guidance in pancreatic cancer. (A) Noninvasive screening for early detection leverages salivary and fecal microbial signatures, including enrichment of *Streptococcus* and *Leptotrichia* in saliva, and Proteobacteria in feces, which demonstrate moderate to high diagnostic accuracy for risk stratification in high‐risk populations (e.g., familial pancreatic cancer, chronic pancreatitis). (B) Invasive precision diagnosis utilizes biliary microbiome profiling from ERCP‐obtained bile samples, which can distinguish malignant pancreaticobiliary obstruction from benign disease based on unique bacterial signatures, enabling complementary diagnostic confirmation in patients undergoing clinical evaluation for obstructive jaundice. (C) Prognostic assessment and longitudinal monitoring incorporate a validated microbial risk score (MRS) integrating 11 survival‐associated bacterial taxa, which stratifies patients into distinct survival groups with a threefold difference in overall survival. Taxa including *Acinetobacter lwoffii* are validated markers of poor prognosis, while beneficial taxa such as *Lactobacillus* and *Escherichia coli* correlate with favorable long‐term survival and enhanced treatment response. Longitudinal monitoring of dynamic changes in these microbial signatures further enables real‐time assessment of treatment response and disease progression.

16S rRNA sequencing reveals that fecal microbial signatures—such as enriched *Proteobacteria*, *Synergistetes*, and *Euryarchaeota*—can distinguish PC patients from healthy controls with high accuracy, even in asymptomatic individuals, positioning them as viable non‐invasive biomarkers for early detection [24, 37, 225, 253, 254, 255]. A fecal microbiota signature identified by Kartal et al. shows high specificity for PC, with potential utility in screening high‐risk populations such as individuals with familial PC or chronic pancreatitis [37].

The bile microbiome also exhibits distinct profiles in PC patients compared to benign pancreaticobiliary diseases [87, 256]. PC patients show lower abundance of *Escherichia* and increased *Streptococcus* in bile samples, providing another potential source of diagnostic biomarkers [87]. Bile samples are obtained via endoscopic retrograde cholangiopancreatography (ERCP), a procedure already used in the evaluation of pancreaticobiliary diseases, making this a clinically feasible approach for differential diagnosis in patients with obstructive jaundice [85].

Oral and salivary microbial signatures offer noninvasive diagnostic potential for large‐scale screening [67, 257, 258]. Salivary profiles of PC patients are characterized by enrichment of *Streptococcus* and *Leptotrichia* and depletion of *Veillonella* and *Neisseria* [257]. These taxa distinguish PC from healthy controls with moderate diagnostic accuracy (AUC values ranging from 0.7 to 0.8). Performance improves when microbial signatures are combined with CA19‐9 or clinical symptoms, though no prospective study has yet demonstrated that this combined approach outperforms CA19‐9 alone in a screening population [259]. Oral samples are easily accessible and can be collected in primary care settings, making them ideal for population‐wide screening efforts [23, 257]. Most microbial biomarker studies report diagnostic accuracy in isolation without directly benchmarking against serum CA19‐9, the current clinical standard. CA19‐9 has a reported sensitivity of ~79%–81% and specificity of ~82%–90% for symptomatic PC [260], although its performance is substantially lower in early‐stage disease. The salivary microbial signatures described above (AUC 0.7–0.8) do not yet surpass CA19‐9 in head‐to‐head comparisons, and their incremental value beyond CA19‐9 remains unproven. Future studies must incorporate CA19‐9 as a mandatory comparator and report net reclassification improvement (NRI) to establish whether microbial biomarkers add clinically meaningful diagnostic information.

Each microbial biomarker platform serves distinct clinical use cases: salivary and fecal signatures are suited for large‐scale screening of high‐risk populations, while bile microbiome analysis adds value for differential diagnosis in patients already undergoing ERCP for biliary obstruction [37, 87, 261]. This stratification aligns microbial biomarkers with existing clinical workflows, accelerating their potential translation to clinical practice [225, 262, 263].

Despite their promise, the clinical feasibility of these salivary and fecal signatures is challenged by inter‐cohort variability and sampling bias [264]. Microbiome profiles are highly sensitive to methodological differences in DNA extraction, sequencing platforms, and bioinformatics pipelines [265]. Furthermore, baseline microbial variations across different geographic populations and diets mean that signatures validated in one cohort may lack reproducibility in another, necessitating standardized, multi‐center validation before clinical adoption [266].

### Prognostic biomarkers

The intratumoral microbiome is strongly associated with prognostic outcomes in PC, providing valuable information for risk stratification and treatment planning [43, 52]. Patients with a high abundance of beneficial taxa such as *Bifidobacterium* and *Lactobacillus* exhibit longer progression‐free survival and overall survival, likely due to enhanced antitumor immunity [221, 262, 267]. These taxa promote M1 macrophage polarization, CD8^+^ T‐cell activation, and reduced inflammation, creating a TME that is less conducive to tumor progression [220, 221, 267, 268]. Conversely, enrichment of pathobionts such as *Fusobacterium* and *Pseudomonas* correlates with poor prognosis, increased therapeutic resistance, and advanced disease stage [33, 196]. *Pseudomonas* species promote chemoresistance by inactivating gemcitabine and enhancing cancer cell survival, further worsening outcomes [196, 269, 270].

Recent large‐scale studies have validated the prognostic potential of microbial signatures [43, 271]. Meng et al. [30] analyzed 201 early‐stage, resected PC patients from the TCGA and ICGC cohorts and identified 11 bacterial species associated with overall survival. These include *Acinetobacter lwoffii*, *Pseudomonas luteola*, and *Shigella flexneri* (poor prognosis) and *E. coli*, *Afipia broomeae*, and *Hymenobacter sp. IS2118* (favorable prognosis). A microbial risk score (MRS) based on these taxa was associated with roughly a threefold difference in overall survival (HR = 2.96, 95% CI: 2.26–3.86, *p* < 0.001). This prognostic value remained consistent across independent cohorts, including after stratification by adjuvant therapy [30]. This MRS also correlates with immune infiltration patterns, including reduced memory B‐cells and increased infiltration of naïve CD4^+^ T cells. Critically, whether the MRS provides independent prognostic value beyond CA19‐9 (the established clinical benchmark) has not been demonstrated. Multivariable analyses incorporating CA19‐9, tumor stage, and treatment regimen are essential to validate the MRS as a clinically useful tool rather than a correlated surrogate of known prognostic factors.

PC patients can be stratified into two microbiome‐related subtypes, Mcluster A and B, with Mcluster B associated with worse prognosis, higher TNM stage, and higher pathological grade. Mcluster B is characterized by enrichment of pro‐inflammatory taxa and reduced microbial diversity, while Mcluster A exhibits higher abundance of beneficial microbes and increased immune infiltration. These subtypes may help guide treatment decisions, as Mcluster A patients are more likely to respond to immunotherapy [272].

## 6 MICROBIOTA‐TARGETED THERAPEUTIC SOLUTIONS

As summarized in Figure 6, a comprehensive framework for precision, microbiota‐targeted interventions—ranging from targeted microbial ablation to synthetic biology‐based approaches—offers a promising roadmap for overcoming current therapeutic barriers. The diverse landscape of microbiota‐targeted therapeutic interventions, ranging from precision ablation and ecological restoration to synthetic biology and metabolic modulation, is further consolidated in Table 2.

**Figure 6:**
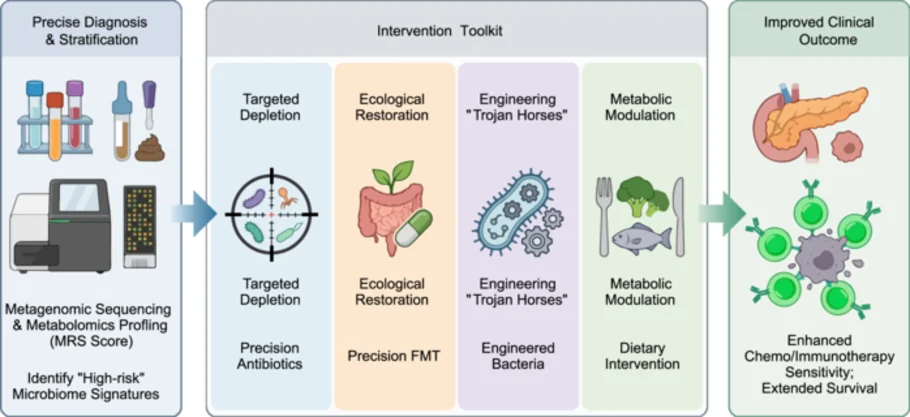
Toward personalized microbiome‐based interventions in pancreatic cancer. Metagenomic sequencing and metabolomic profiling are first used to identify high‐risk microbiome signatures and enable patient stratification. Based on these profiles, a targeted intervention toolkit is applied. This includes targeted depletion using precision antibiotics, such as ciprofloxacin selectively targeting γ‐proteobacteria, or bacteriophage therapy. The toolkit also involves ecological restoration through fecal microbiota transplantation (FMT) from long‐term survivors or defined protective consortia comprising protective *Bifidobacterium* strains. Furthermore, it incorporates engineering “Trojan horse” strategies employing genetically modified bacteria to locally deliver immunomodulatory agents, including IL‐12 and anti‐PD‐L1 nanobodies. Finally, the approach integrates metabolic modulation via dietary interventions, including tryptophan‐rich diets to increase 3‐IAA production and adherence to a Mediterranean diet, which are designed to alter microbial metabolite production. These microbiome‐informed strategies aim to enhance sensitivity to chemotherapy and immunotherapy, ultimately improving clinical outcomes.

**Table 2:** Summary of microbiota‐targeted therapeutic strategies.

Strategies	Interventions	Representative targets/mechanisms	Clinical objectives
Precision antibiotics	Ciprofloxacin	Targeting γ‐proteobacteria	Restore gemcitabine sensitivity
Precision antibiotics	Metronidazole	Targeting anaerobes	Enhance anti‐PD‐1 efficacy
Probiotics	*Clostridium butyricum*	Inducing ferroptosis via butyrate production	Synergize with chemo; reduce GI toxicity
FMT	Targeted donor FMT	Long‐term survivors or ICI responders	Remodel TME; overcome resistance
Synthetic biology	Engineered *E. coli*	Localized IL‐12 release	Activate intratumoral T‐cell infiltration
Synthetic biology	Engineered BEVs	siRNA delivery against bacterial CDA	Block microbial drug inactivation
Metabolic modulation	Dietary intervention	Time‐limited tryptophan supplementation	Optimize the microbiome‐metabolite axis

Abbreviations: CDA, cytidine deaminase; FMT, fecal microbiota transplantation.

### Precision antibiotics

Precision antibiotics target specific protumorigenic taxa responsible for therapy resistance while preserving overall microbial balance [273, 274, 275]. Ciprofloxacin, a fluoroquinolone that selectively targets γ‐proteobacteria, has been shown to restore gemcitabine sensitivity in murine models by inhibiting cytidine deaminase‐expressing taxa [196]. However, as a broad‐spectrum antibiotic, it may disrupt commensal gut microbiota, and its use requires balancing the benefit of salvaging gemcitabine efficacy against the risk of dysbiosis‐induced immunosuppression. Emerging clinical evidence suggests that antibiotics targeting Gram‐negative bacteria may improve gemcitabine efficacy in PC patients with high intratumoral bacterial burden, but only in a personalized setting [276]. This personalized approach ensures that only patients likely to benefit from antibiotic co‐administration receive treatment, minimizing off‐target effects [42, 196]. Metronidazole is a nitroimidazole antibiotic that selectively targets anaerobic bacteria, including *F. nucleatum* and *P. gingivalis*, which have been implicated in immunotherapy resistance [139, 274, 277]. Preclinical models demonstrate that metronidazole‐mediated depletion of these taxa restores CD8^+^ T‐cell infiltration and enhances anti‐PD‐1 efficacy [114, 277, 278]. Ongoing early‐phase clinical trials are testing metronidazole in combination with ICIs for PC patients with high anaerobic microbial abundance, with early data indicating improved immune activation within the TME [279].

Novel precision platforms such as phage therapy and targeted antimicrobial peptides are under preclinical investigation for selectively eliminating protumorigenic taxa [28, 81, 280]. Phages may be engineered to target specific bacteria, while antimicrobial peptides can be designed against unique bacterial cell wall components [46]. However, no phage therapy or antimicrobial peptide has entered clinical trials for PC, and substantial barriers in delivery, specificity, and safety remain to be addressed [81, 281].

### Probiotics and prebiotics for therapeutic enhancement

Probiotics and prebiotics are designed to restore microbial balance, enhance beneficial microbial activity, and counteract the pro‐tumorigenic effects of dysbiosis [19, 20, 107, 267, 282]. Unlike conventional probiotic formulations, those tailored for PC are selected based on their ability to directly address the specific mechanisms of treatment failure [19, 81, 107, 283].

Specifically, *C. butyricum* supplementation has been shown to reduce chemotherapy‐induced gastrointestinal toxicity while enhancing drug efficacy via previously discussed ferroptosis mechanisms [149, 284]. Similarly, to overcome immunotherapy resistance, *B. pseudolongum* is currently being evaluated in clinical trials in combination with anti‐PD‐L1 therapy, leveraging its ability to produce immunomodulatory metabolites like inosine and shift macrophage polarization to restore T‐cell function [24, 221, 284, 285, 286].

Prebiotics such as fructooligosaccharides and inulin are used to selectively promote the growth of beneficial probiotic strains [183, 287, 288, 289]. Fructooligosaccharides preferentially support *Bifidobacterium* and *Lactobacillus* species, increasing short‐chain fatty acid production and enhancing gut barrier function [109, 287, 290, 291, 292]. In murine models, prebiotic supplementation enhances gemcitabine efficacy by reducing intratumoral γ‐proteobacteria abundance and promoting antitumor immunity [183, 196, 289]. Combined with probiotics, prebiotics create a synergistic effect that modulates the gut‐pancreatic microbiome axis to overcome treatment resistance [81, 109, 287, 289, 293, 294]. Postbiotics, defined as soluble metabolites derived from probiotics, have emerged as a potentially more stable alternative to live probiotic administration [295, 296]. Butyrate, a key postbiotic, enhances CD8^+^ T‐cell cytotoxicity via GPR109A‐HOPX signaling, while *Lactobacillus*‐derived peptides reduce inflammation and tumor burden [297, 298, 299]. These postbiotics can be administered in standardized doses, avoiding the challenges of microbial engraftment associated with live probiotics [300, 301, 302].

### FMT for precision immunomodulation

FMT is a powerful tool to restore eubiosis by transferring a healthy, diverse microbial community from a donor to a PC patient [42, 303, 304]. Unlike conventional FMT approaches, precision FMT for PC targets the specific microbial deficiencies driving treatment resistance, using donors selected based on their microbial composition and functional profile [42, 263, 304, 305].

Donor selection is critical for FMT success. Ideal donors exhibit high microbial diversity, low abundance of pro‐tumorigenic taxa, and high levels of beneficial taxa (e.g., *Bifidobacterium*, *A. muciniphila*) [42, 304]. Donors may also be selected based on their clinical response to PC therapies, as FMT from ICI‐responsive donors has been shown to transfer therapeutic responsiveness to germ‐free mice [39, 221, 306]. Personalized FMT, using donors with microbial signatures that complement the patient's dysbiosis profile, optimizes outcomes by directly addressing the patient's specific microbial deficiencies [263, 304, 307, 308].

Clinical trials of FMT in PC are ongoing, evaluating its safety and efficacy in combination with ICIs and chemotherapy [107, 263, 304]. A phase I trial of oral FMT capsules (LND101) in advanced PC patients is testing whether FMT can enhance immune infiltration in the TME and improve treatment response [309]. Early data suggest that FMT is well tolerated and leads to microbial engraftment, with preliminary signs of improved immune activation [247, 310]. FMT may also be used to reverse antibiotic‐induced dysbiosis in PC patients [42, 304, 310, 311]. Broad‐spectrum antibiotic use during chemotherapy disrupts beneficial microbes, and FMT can restore microbial diversity and function, enhancing subsequent treatment efficacy [312, 313]. In combination with precision antibiotics, FMT allows sequential depletion of pro‐tumorigenic taxa and restoration of beneficial microbes to sustain eubiosis and antitumor immunity [107, 311].

### Engineered microbes for targeted therapy

Advances in synthetic biology have enabled experimental engineering of microbes for potential cancer therapy, though all current approaches remain at preclinical stages [314, 315, 316, 317]. In preclinical models, engineered *E. coli* producing IL‐12 in the TME activates dendritic cells and enhances CD8 + T‐cell infiltration, reversing immunosuppression and enhancing ICI efficacy [318, 319, 320, 321, 322]. Engineered *Salmonella typhimurium* selectively colonizes hypoxic tumor regions to deliver immunostimulatory molecules [321, 322]. However, clinical translation faces major hurdles including tumor colonization efficiency, host immunogenicity, and biosafety concerns (see Section 7). BEVs have also been engineered as targeted delivery vehicles [323, 324, 325, 326]. *E. coli* outer membrane vesicles expressing PD‐1 bind to PD‐L1 on tumor cells, blocking the immunosuppressive PD‐1/PD‐L1 axis and enhancing T‐cell activation. Engineered BEVs carrying siRNA against cytidine deaminase reduce gemcitabine inactivation by γ‐proteobacteria, restoring drug efficacy [313, 325, 327]. These engineered BEVs provide a precise method to target microbial‐mediated resistance without disrupting the overall microbial community.

### Dietary interventions to modulate the microbiome‐therapy axis

Dietary strategies complement other microbiota‐targeted therapies by shaping the gut‐pancreatic microbiome axis to enhance treatment efficacy [328, 329, 330]. The timing of tryptophan supplementation is critical: short‐term administration enhances chemotherapy efficacy via the 3‐IAA/MPO axis, while prolonged intake may promote pro‐tumorigenic inflammation, underscoring the need for personalized, time‐limited interventions [24, 51, 331, 332].

The Mediterranean diet, rich in prebiotic fibers, fermented foods, and healthy fats, modulates the gut microbiome to reduce inflammation and enhance antitumor immunity [111, 333]. This diet promotes the growth of beneficial microbes such as *Bifidobacterium* and *Lactobacillus*, increasing short‐chain fatty acid production and improving gut barrier function [110, 111, 334]. In PC patients, adherence to the Mediterranean diet correlates with improved chemotherapy tolerance and longer survival, making it a cost‐effective adjunct to conventional therapy [335]. Dietary restriction of choline and l‐carnitine reduces trimethylamine N‐oxide production, mitigating its pro‐tumorigenic effects on pancreatic metabolism and inflammation [336, 337]. This intervention is particularly beneficial for patients with a high abundance of TMAO‐producing gut bacteria, helping to reverse metabolic reprogramming and stromal remodeling [328]. These dietary strategies are not one‐size‐fits‐all but are personalized based on the patient's microbial profile, ensuring that interventions directly address their specific microbial‐mediated treatment barriers.

## 7 CHALLENGES AND FUTURE DIRECTIONS

### Methodological standardization

Standardization of microbial profiling remains a major barrier to advancing microbiome‐driven PC research [46, 253, 337]. Current studies vary widely in sample collection, DNA extraction protocols, sequencing platforms, and contamination control strategies, resulting in inconsistent taxonomic and functional annotations [253, 338]. Fresh tissue samples yield higher‐quality microbial DNA compared to formalin‐fixed paraffin‐embedded (FFPE) samples, but FFPE samples are more readily available in clinical settings [253, 339, 340]. Differences in DNA extraction methods can introduce bias in microbial community profiling, as some protocols are more effective at extracting DNA from Gram‐positive bacteria or fungi [341, 342]. Low‐biomass contamination in pancreatic tumor samples presents a particularly pervasive challenge, discussed in detail below. Pancreatic tissue harbors a microbial biomass several orders of magnitude lower than that of the gut or oral cavity, making contamination from environmental sources, laboratory reagents, and sample handling a pervasive and underappreciated problem [40, 343, 344]. Low‐biomass samples are particularly susceptible to false‐positive signals, as contaminant DNA from extraction kits, PCR reagents, and laboratory surfaces can dominate the sequencing output, obscuring true microbial signatures [343]. This issue is especially acute for PC studies, where tissue is often obtained through endoscopic procedures or surgical resections that introduce additional contamination risks.

Several best practices are recommended to mitigate contamination. First, inclusion of negative controls (blank DNA extractions, swab controls from surgical instruments, and environmental air controls) at every step from sample collection through sequencing is essential [345, 346]. Second, computational decontamination tools such as Decontam and SCRUB can statistically identify and remove likely contaminant sequences based on their frequency patterns and prevalence in negative controls [347]. Third, quantitative approaches including droplet digital PCR and spike‐in controls with known microbial loads can verify that detected taxa exceed background levels [348]. Fourth, independent validation cohorts using orthogonal methods (e.g., FISH, culture‐based approaches) are necessary to confirm sequencing‐based findings. The field must move toward consensus guidelines for low‐biomass microbiome research in PC, analogous to the MIQE guidelines for qPCR or the STROBE statement for observational studies. Until such standards are widely adopted, the reproducibility of pancreatic microbiome studies—and the clinical biomarkers derived from them—will remain uncertain. Future studies should report contamination metrics transparently and include contamination assessment as a standard component of study quality evaluation.

Beyond technical standardization, future efforts should prioritize moving beyond genus‐level taxonomic classification to functional genomic characterization: isolating microbial strains directly from PC tissues and patient samples, followed by complete genome sequencing to resolve intraspecies heterogeneity. This addresses the critical limitation that genus‐level identification (e.g., *Pseudomonas* or *Bacteroides*) lacks biological informativeness—for example, distinguishing pathogenic *Pseudomonas* (a soil/water bacterium prone to clinical sample contamination) from non‐pathogenic *Pseudomonas* species, or toxin‐producing subtypes of *B. fragilis* relevant to PC oncogenesis. Integrating AI‐driven genomic analysis with multi‐omics data will further enable the identification of PC‐associated bacterial/fungal genes and functional modules.

The Microbiome Quality Control consortium has established guidelines for fecal microbiome profiling, and similar frameworks are needed for pancreatic tumor samples, analogous to the quality control frameworks established for The Cancer Genome Atlas in somatic mutation research [338, 349].

### Validation and generalization of microbial signatures

Most microbial biomarkers and functional mechanisms identified to date are based on small cohorts or preclinical models, lacking validation in large, heterogeneous patient populations [46, 338, 340]. Microbial risk scores and subtype‐specific signatures show promise for prognosis, but their utility across diverse ethnicities, geographic regions, and treatment backgrounds remains untested [19, 42, 263]. Host‐microbe interactions are highly context‐dependent, with factors such as age, sex, smoking status, and diet modulating microbial composition and function [116, 350]. Few studies integrate these variables into predictive models, limiting their clinical applicability.

Transitioning from correlative observations to causal evidence remains the central challenge in pancreatic microbiome research. Most published findings to date are associative, derived from cross‐sectional sequencing studies with limited experimental validation. The field must prioritize mechanistic studies using germ‐free models, microbial reconstitution, and single‐cell functional readouts to move beyond description toward causality. Specifically, future investigations should prioritize whether cross‐tumor conserved mechanisms—such as Gal‐GalNAc binding pathways—are functionally active within the pancreatic stroma [351]. Moving beyond descriptive taxonomy, it is equally imperative to decode the specific oncogenic mechanisms of understudied species like *G. morbillorum* [225, 352]. Furthermore, characterizing the microbial landscape of liver metastases in parallel with primary tumors will be essential to identify potential ‘metastasis‐associated’ signatures that may drive systemic disease progression [353]. By integrating germ‐free models with single‐cell resolution, the field can effectively distinguish true microbial ‘drivers’ of malignancy from opportunistic “passengers” colonizing the altered tumor microenvironment.

Large‐scale, multi‐center prospective cohorts are necessary to validate microbial signatures against CA19‐9 as the established clinical benchmark, disentangle confounding factors, and establish whether microbial biomarkers add independent diagnostic or prognostic value beyond standard‐of‐care tools [19, 263, 350]. These cohorts should include diverse patient populations to ensure generalizability, and longitudinal sampling should be conducted to track changes in the microbiome during disease progression and treatment [40, 42, 271]. International collaborations such as the International Cancer Microbiome Consortium are working to address this gap by aggregating data from multiple studies and standardizing analytical pipelines [253, 354]. Furthermore, the investigation of tumor microenvironment interactions in situ can be significantly enhanced by advanced imaging approaches. For instance, comparing tumor‐nonspecific and PD‐L1‐specific imaging via near‐infrared fluorescence and Cerenkov luminescence dual‐modality in situ imaging provides valuable spatial insights into tumor‐associated biomarkers, which could be adapted to visualize host‐microbiome interactions in real time [355].

### Expanding beyond bacteria: Prioritizing mycobiome and virome research

The current focus on bacterial communities overlooks the potential contributions of fungi and viruses to PC biology [40, 45, 66]. The mycobiome, dominated by *Candida* species, has been associated with carcinogenesis and immune modulation, though causal evidence in PC remains limited [25, 45, 356, 357]. Recent evidence further implicates *Malassezia* spp. in PC progression through the activation of the MBL pathway, C3 complement cascade, and IL‐33/ILC2‐mediated type 2 immunity, thereby highlighting the need for targeted functional studies of non‐*Candida* fungal taxa to prevent an incomplete understanding of the mycobiome [20, 45, 58, 358].

Viruses and bacteriophages may modulate bacterial communities or directly interact with cancer cells, but technical limitations in sequencing low‐abundance viral taxa have hindered progress [61, 345, 346, 359]. Future research must prioritize comprehensive multi‐kingdom profiling, integrating bacterial, fungal, and viral data to capture the full complexity of the intratumoral microbiome. Advances in metagenomic sequencing and bioinformatic tools are enabling deeper characterization of these non‐bacterial components, although additional studies are needed to validate their functional roles [19, 45]. Targeted studies of the mycobiome and virome may uncover novel therapeutic targets and biomarkers, addressing a critical gap in current PC microbiome research [28, 58, 271].

Distinguishing causal microbial drivers from correlative passengers remains a key challenge [46, 360]. While preclinical models such as germ‐free mice and FMT provide strong evidence for causality, translating these findings to humans is complex [361, 362]. Future studies should employ complementary approaches to establish causality, including targeted microbial ablation, metabolite supplementation/inhibition, and single‐cell sequencing to map microbe‐host interactions at the cellular level [24, 31, 361].

As shown in Figure 7, integration of multi‐omics data, including metagenomics, transcriptomics, metabolomics, and immunomics, enables a systems‐level understanding of microbiome‐mediated mechanisms [43, 363]. This holistic approach can identify key microbial metabolites, signaling pathways, and immune cell interactions that drive PC progression and treatment resistance, providing a more robust foundation for therapeutic development.

**Figure 7:**
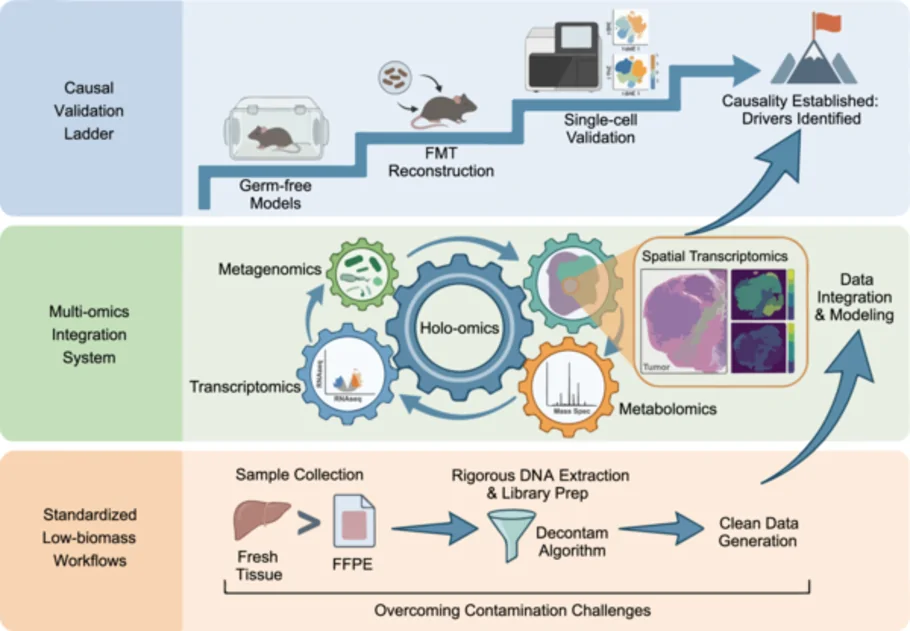
Methodological framework for establishing causal microbiome–tumor interactions in pancreatic cancer. Standardized low‐biomass workflows are implemented to minimize contamination, encompassing optimized sample collection from fresh and formalin‐fixed paraffin‐embedded (FFPE) tissues, rigorous DNA extraction and library preparation, and computational decontamination algorithms to enable clean data generation. Multi‐omics integration combines metagenomics, transcriptomics, metabolomics, and spatial transcriptomics within a holo‐omics framework, followed by data integration and modeling to resolve tumor–microbiome interactions at high spatial and cellular resolution. Causal validation is achieved through a stepwise validation ladder, including germ‐free models, fecal microbiota transplantation (FMT) reconstruction, and single‐cell–level functional validation, ultimately enabling the identification of microbial drivers with causal roles in tumor progression.

Despite promising preclinical results, translating microbiome‐targeted strategies into effective PC treatments faces significant hurdles [106, 364]. The lack of personalized approaches limits efficacy, as microbial signatures vary widely between patients [31, 272]. A one‐size‐fits‐all therapy, such as non‐selective probiotics, may not benefit all individuals; patient‐specific microbial profiling is therefore needed to guide treatment selection [365, 366].

Safety concerns persist, particularly for FMT and broad‐spectrum antibiotics. For FMT, engraftment failure is a major issue—PC patients often have disrupted gut environments that resist new microbial colonization. For engineered microbes, tumor colonization efficiency remains a bottleneck, as insufficient bacteria reach the tumor site after systemic administration. FMT also carries risks of pathogen transmission and immune‐related adverse events, while broad‐spectrum antibiotics can disrupt beneficial microbes and exacerbate dysbiosis [109, 367, 368]. The optimal timing, dosing, and combination of microbiome therapies with chemotherapy, immunotherapy, or targeted therapy remain undefined [46, 106, 109, 369]. For example, while FMT from ICI‐responsive donors enhances anti‐PD‐1 efficacy in preclinical models, clinical trials must address donor selection, delivery routes, and compatibility with existing treatments [42, 46, 106, 109, 367, 370].

Substantial regulatory and safety challenges also exist for engineered microbial therapies [81, 370]. Because these therapies are classified as biological products involving live, genetically modified organisms, their development requires rigorous safety and efficacy testing [371, 372]. The potential for severe host immunogenicity, including the risk of cytokine storms, immune clearance of the microbes before reaching the tumor, and unintended off‐target genetic transfers must be rigorously addressed. Furthermore, standardized manufacturing processes and integrated failsafe mechanisms, including inducible kill switches, are urgently needed to ensure consistency and quality control [371, 373].

## 8 CONCLUSIONS

The intratumoral microbiome has emerged as a central regulator of PC initiation, progression, and therapeutic response, offering novel avenues for diagnosis, prognosis, and treatment. By distinguishing between microbial‐mediated problems and targeted solutions, this review provides a structured framework for understanding how microbial communities undermine treatment efficacy and how precision microbiota‐targeted strategies can overcome these barriers. The integration of fungal and viral components, where evidence permits, along with explicit attention to causal inference, addresses key gaps in the current literature while acknowledging the preliminary nature of non‐bacterial microbiome research in pancreatic cancer.

While significant progress has been made in characterizing microbial composition and defining functional mechanisms, critical challenges in standardization, validation, and clinical translation remain. Addressing these obstacles will require interdisciplinary collaboration across microbiology, oncology, immunology, and bioinformatics, as well as large‐scale prospective studies and innovative clinical trials.

As research advances, integrating microbial insights into PC care holds great promise for improving early detection, personalizing treatment strategies, and overcoming therapeutic resistance. By systematically unraveling the complex crosstalk between intratumoral microbes, host cells, and the immune system—and rigorously distinguishing causal mechanisms from correlative observations—the field may develop microbiome‐informed therapies that enhance anti‐tumor immunity and improve patient outcomes. Over the next decade, microbiome research is poised to transition from preclinical discovery to clinical application, heralding a new era in the management of this lethal malignancy.

## AI STATEMENT

Gemini 3 (Google) was used for improving the language and readability of the text. All content has been verified for accuracy by the authors.

## AUTHOR CONTRIBUTIONS

**Yuqin Xie**: Conceptualization, writing—original draft, visualization. **Jingyuan Ye**: Writing—original draft, Investigation. **Sheng Wang**: Writing—original draft, Investigation. **Maoyun Liu**: Writing—review and editing. **Fengsheng Dai**: Writing—review and editing. **Mingzhen Bai**: Writing—review and editing. **Yi Wang**: Writing—review and editing. **Athena Savoji**: Writing—review and editing. **Yu Wang**: Writing—review and editing. **Phillip B. Hylemon**: Supervision, Writing—review and editing. **Huiping Zhou**: Supervision, writing—review and editing. **Hui Li**: Conceptualization, supervision, funding acquisition, writing—review and editing. **Dewei Li**: Conceptualization, supervision, funding acquisition, project administration, writing—review and editing. All authors have read the final manuscript and approved it for publication.

## CONFLICT OF INTEREST STATEMENT

The authors declare no conflicts of interest.

## ETHICS STATEMENT

No animals or humans were involved in this study.

## Data Availability

No new datasets were generated or analyzed during the current study. Supplementary materials (graphical abstract, slides, videos, Chinese translated version and update materials) may be found in the online DOI or iMeta Science http://www.imeta.science/imetaomics.
